# Peculiarities of the Lesions of Spavin

**Published:** 1898-04

**Authors:** S. J. J. Harger

**Affiliations:** Professor of Veterinary Anatomy and Zoötechnics, University of Pennsylvania


### PECULIARITIES OF THE LESIONS OF SPAVIN.

By S. J. J. Harger, V.M.D.,
PROFESSOR OF VETERINARY ANATOMY AND ZOÖTECHNICS, UNIVERSITY OF PENNSYLVANIA.

The discussion of the disease of the hock, ordinarily called bone-spavin, is not new, and if I only repeat old things, I am repaid if the subject is presented to some of you in a new light.

The anatomic construction of the hock renders it somewhat difficult to understand the precise nature of some of its pathologic conditions. The hock-joint overcomes at each step a very great resistance, a work which it could not accomplish were it not for the mechanical arrangements of the bones, ligaments, fibrous bands, and tendons. In performing its function it acts as a lever of the second class, the arm of the lever being the tarsal bones and the canon from the point of the hock to the fetlock; the power is applied at the point of the hock; the weight to be displaced forward in locomotion is exerted upon the astragalus by the lower end of the tibia; the fetlock is the fulcrum. On account of the intimate union of the hock-bones and the latter to the canon- and splint-bones, the bony ray from the point of the hock to the fetlock may for the time be considered as one rigid piece. Toward the upper extremity, at the astragalus and the lower extremity of the tibia, it supports the weight of the member, under which weight its tendency is to give or break at some point. The strain on this rigid rod of bone, its theoretical tendency to bend, and the consequent overstretching or rupture of some of its ligaments, are greater the more nearly perpendicular the tibia is to the back, because then the perforans, perforatus gastrocnemius, and tibial fascia are more powerful, since their angle of attachment to the bones tends toward a right angle. Thus the integrity of the hock-joint (not including lateral displacement) is in greatest danger when partially flexed. This is seen momentarily when the foot touches the ground and before the leg is straightened to throw the body forward ; also in slipping and trying to recover from a misstep ; that is, at the beginning of the phase of contact.

The part that yields first is the one most frequently diseased. All know that it is the inner and lower parts of the hock which are most frequently diseased, and the study of the mechanism of this joint is necessary in order to interpret all the facts. These parts suffer most because the bones and ligaments of this region, relatively or absolutely, support more weight or pressure, traction and concussion than those in the other parts. Among such predisposing conditions may be mentioned: 1. Greater development and strength of the hock-bones on the outside; a vertical line passing through the centre of the canon-bone divides the hock into two unequal portions, the internal being the smaller. 2. The obliquity of the tibia. This bone slants downward and inward by its lower extremity, and aided by the obliquity of the articular surface and the prominence of the internal lip tends to press downward, but a little inward, to the inner side. 3. The line of vertical pressure of the tibia upon the hock-bones. This pressure is received upon the pulley of the astragalus, the greater portion of which is carried in a more vertical line to the scaphoid, two cuneiforms, and the inner half of the canon and internal splints, which are directly superposed. On the outside this condition does not exist, and much of the force is destroyed before it reaches these bones, which do not lie in the direct line of pressure of the tibia upon the astragalus, but rather to the outside.

The line of greatest pressure in the hock from above to below seems to slant obliquely inward to the lower end of the internal side. This is indicated by the inward slant of the articular surfaces of the scaphoid, cuboid, and canon-bones. It may be presumed that if the pressure were equally distributed, these surfaces would be more horizontal. These conditions are sometimes made more effective by a faulty external conformation, and thus predispose to spavin.

Opinions are at variance as to the seat of the initial lesions and the order in which the alterations follow. Diekerhoff's theory of the cunean tendon can be dismissed after reasoning from the manner of attachment of that tendon, and the fact that inflammation of the synovial bursa is so rare, and when it does exist causes no lameness. Again, according to some writers, spavin commences as a periostitis due to a hyperextension of the lateral ligaments, and in their opinion bony enlargement is primary; others maintain that the articular surfaces are primarily affected and that the enlargement is secondary. Both views seem to have equally authoritative advocates, although the former is the one most generally believed.

I have had at my command between forty and fifty specimens of spavined hocks, which I have carefully examined in order to endeavor to make any possible deductions as to the point of the beginning of the disease and the order of succession of the various lesions, and the order in which the steps of the alterations follow each other. But the primary seat seems to have been constant—that is, the initial lesion of a chronic spavin appears to be a chronic arthritis of the hock-bones affecting the articular surfaces between two adjoining bones, with desquamation of the articular cartilage, etc. This process is the first stage in the development of spavin. In the second stage the ostitis terminates in a coössification of these diseased bones. In the third stage the articular inflammation spreads to the periphery of the bone, to the periosteum, causing a periostitis with a bony enlargement. If the diseased process becomes generalized, these various phases are progressing simultaneously in different parts of the articulation. As evidence of chronic arthritis, some of the bones were always found, and with unexpected frequency, without bony deposit on the outside of the bone. The bony deposit on the outside, unless it was very small, was seen only after the coössification of the bones of the lower row with one another and with the metacarpals was well advanced. I can only speak from what I have observed; but it seemed to me that the earliest and most frequent bones to coössify were the scaphoid and large cuneiform, and the cuneiforms to the metatarsus in decreasing order. The small cuneiforms did not show any evidence of being so often primarily affected, contrary to what is usually said. A bone tumor was not observed unless the union of the bones implicated was completed, excepting a small bony deposit at the upper end of the canon and in front, due to a laceration of the tendons of the flexor metatarsi. I do not mean to intimate that all bony enlargements of the internal side of the hock are secondary to articular lesions, and that the reverse cannot be true. I only refer to the average chronic spavin as one usually sees it. Such enlargements may result from a primary periostitis, from laceration of the peripheral ligaments, due to violent traumatisms of the hock, resulting in a generalized periostitis. Accepting this view, we can explain how deep-seated the cause of lameness may be without any outward signs, and how difficult the diagnosis may be.

We have often had cases of lameness which we were inclined to locate in the hock, but perhaps we hesitated because of the absence of local symptoms. The local fever is sometimes well marked; but it appears to me that in mild lesions its absence would not be negative proof. The lameness may precede the presence of an enlargement for some time. This period may be variable. In one instance, on reliable information, a mare was lame for a year from a suspected spavin, and at the autopsy complete anchylosis of one hock was found. In one instance under my observation, a mare was lame for three months, presenting the symptoms indicative of hock-lameness, but there was no difference between the hocks, excepting a decided fever on the internal side of the one that was diseased. Other cases of obscure lameness are met in practice which may be placed under this category while, at the same time, in the absence of other symptoms, our opinion should be guarded.

The term "acute" spavin applies, then, only to a desquamation or the first stage of an ordinary spavin, which may be of variable duration. The articular cartilage when once desquamated and absorbed, not being able to regenerate itself, we can readily understand that the treatment employed in such cases is not of use because it is counter-irritant, but must always be directed toward hastening the anchylosis.

The lesion of spavin is, therefore, exactly the opposite to that of ringbone—a difference dependent upon different anatomical conditions. In the latter (ringbone), in all the specimens which I have examined, the primary lesion is a periostitis and a peripheral deposit of bone. It is only in the very last stages that the articular cartilage of the phalanges becomes destroyed and anchylosis takes place; in fact, the bony deposit may be very large and the mechanical interference with the articulation very considerable, and still the articular surfaces be intact. The latter being so large and freely movable, making anchylosis with artificial means very difficult, combined with the mechanical interference of the movements, explains the poor success obtained in treating this diseased condition.

From a legal standpoint it may be said that the simple coössification of the lower bones of the hock is not incompatible with apparent soundness in the gait. We know that there is a natural tendency in the horse's foot for some of the bones to atrophy and become coössified with others, such as the canon- and splint-bones. His manner of locomotion requires this. The same is true of the hock-bones. Some of them may coössify without any external alterations and without at any time showing any lameness, the animal having been always under observation. The anchylosis in both hocks is alike. This seems to be due to a constitutional predisposition, and during this process an exciting cause less powerful than under ordinary conditions may determine a spavin.

